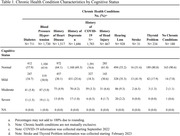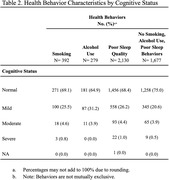# Mobile Early Detection Memory Screening in the Republic of Armenia

**DOI:** 10.1002/alz.086148

**Published:** 2025-01-09

**Authors:** Jane Mahakian, Arina Megerdichian, Pateel Jivalagian, Tatjana Novakovic – Agopian, Armen Moughamian, Joan Monin

**Affiliations:** ^1^ Alzheimer’s Care Armenia, San Clemente, CA USA; ^2^ University of Southern California, Los Angeles, CA USA; ^3^ Yale University School of Public Health, New Haven, CT USA; ^4^ Palo Alto VA Medical Center, Palo Alto, CA USA; ^5^ Ray Dolby Brain Health Center, San Francisco, CA USA; ^6^ Yale School of Public Health, New Haven, CT USA

## Abstract

**Background:**

The Republic of Armenia is a post‐Soviet, low‐ and middle‐income country (LMIC) in the south Caucasus region with a steadily increasing aging population. The goal of this study was to provide the first look into the national cognitive health in Armenia, considering the growing burden of cognitive impairment (CI) and widespread lack of public awareness about dementia. As a component of the early detection memory screening program launched by Alzheimer’s Care Armenia’s Brain Health Project and funded through Davos Alzheimer’s Collaborative (DAC), this study aimed to understand the prevalence of CI and associated factors across the adult population.

**Methods:**

Utilizing a mobile clinic, a sample of 4,066 adults (aged 25‐94) were screened for cognitive impairment across 8 urban and rural provinces in Armenia. Participants completed a Montreal Cognitive Assessment (MoCA) screening test and Health Characteristic Questionnaire including items about health behaviors and chronic health conditions. Statistical analyses were used to investigate demographic trends of CI and test for significant associations.

**Results:**

MoCA scores indicated the following cognitive levels in this population: 71.2% normal cognition, 23.7% mild cognitive impairment, 4.2% moderate cognitive impairment, and 0.8% severe cognitive impairment. The most prevalent chronic conditions included history of COVID, hypertension, history of depression, and history of heart disease (Table 1). The most common health behavior was poor sleep quality (Table 2). All health behaviors and chronic health conditions were significantly associated with CI. The sample consisted of mostly women (81.5%), individuals with 12 or less years of education, higher BMI levels, and those living in rural areas, which may present potential limitations.

**Conclusion:**

Findings reveal lifestyle and environmental exposures relevant to CI and highlight the possible influence of behavioral and cultural factors on dementia development. As the first study to investigate the prevalence of CI and associated factors in Armenia, this research lays the foundations for understanding unmet needs for cognitive health, guiding future policy, and establishing sustainable health infrastructure in similar post‐Soviet, LMIC. Future research should be aimed at further investigating which risk factors are predictive of cognitive status and dementia development in the region.